# Dynamic Coding of Signed Quantities in Cortical Feedback Circuits

**DOI:** 10.3389/fpsyg.2012.00254

**Published:** 2012-08-03

**Authors:** Dana H. Ballard, Janneke Jehee

**Affiliations:** ^1^Department of Computer Science, University of Texas at AustinAustin, TX, USA; ^2^Centre for Cognitive Neuroimaging, Donders Institute for Brain, Cognition and BehaviorNijmegen, Netherlands

**Keywords:** codes, cortex, learning, receptive field, sensory, sparse coding, synapses

## Abstract

In the early sensory and motor areas of the cortex, individual neurons transmit information about specific sensory features via a peaked response. This concept has been crystallized as “labeled lines,” to denote that axons communicate the specific properties of their sensory or motor parent cell. Such cells also can be characterized as being polarized, that is, as representing a signed quantity that is either positive or negative. We show in a model simulation that there are two important consequences when learning receptive fields using such signed codings in circuits that subtract different inputs. The first is that, in feedback circuits using labeled lines, such arithmetic operations need to be distributed across multiple distinct pathways. The second consequence is that such pathways must be necessarily dynamic, i.e., that synapses can grow and retract when forming receptive fields. The model monitors the breaking and growing of new circuit connections when their synapses need to change polarities and predicts that the rate of such changes should be inversely correlated with the progress of receptive field formation.

## Introduction

Most neural sensory circuits are faced with the issue of representing negative quantities and there are different strategies for doing so. One way is to have an individual neuron vary its firing rate: The brainstem circuitry of the vestibulo-occular reflex (VOR) tracks head velocity very precisely with firing rates that vary between 100 and 300 spikes/s (Fuchs and Kimm, 1975). By convention the extreme negative value is 100 spikes/s and the extreme positive value is 300 spikes/s. The logical “zero” is then at 200 spikes/s.

In contrast to the VOR’s spike rate encoding, the cortex uses a very different coding strategy where two neurons represent the different polarities of a quantitative feature, one for positive and one for negative. In their original experiments, Wiesel and Hubel (1966) characterized a cell’s peaked response to a stimulus feature as a *labeled line*, and thus we use the phrase *signed labeled lines* to specifically note that the quantities are part of a two-cell representation for signed numbers. Signed labeled lines are ubiquitous in cat and primate cortex. Simple edge cells, direction-sensitive cells (Orban et al., 1986), opponent color cells (Livingstone and Hubel, 1984), disparity cells (LeVay and Voight, 1988), motion cells (Rodman and Albright, 1987), as well as many more types, all use this coding strategy. However it turns out that this coding strategy poses difficulties for feedback circuits that use subtraction, since the desired circuit depends on the relative magnitudes of the minuend and subtrahend.

This paper introduces a methodology for dealing with signed labeled lines that explicitly represents positive and negative quantities in a way that allows us to demonstrate the consequences of subtraction in bipolar pairs of cells. We model the feedback circuit between the striate cortex and the lateral geniculate nucleus (LGN). The model feedback circuit learns synaptic weights by training itself on appropriately filtered natural image patches (Jehee et al., 2006). The learning algorithm is based on matching pursuit (Mallat and Zhang, 1993), which has a simple geometric interpretation. This general class of algorithms originally modeled the formation of simple cell receptive fields (Olshausen and Field, 1996) and has been subsequently extended to cortical hierarchies (Rao and Ballard, 1999). Its importance is that it does not specify connections in detail but instead relies on a general abstract principle that the synapses should be chosen to minimize the number of active neurons that are need to code any particular input pattern. The predictive coding approach, in a slightly different form that uses competition and divisive normalization, has been shown to account for an impressive array of experimental observations (Spratling, 2008, 2010, 2012).

Our concern has focused on extending the (Jehee et al., 2006) algorithm to a lower level of abstraction that could account for computation by individual spikes. In Jehee and Ballard (2009), we showed that the predictive coding could model the rebound effects observed in the reverse correlation of LGN cell responses. In Ballard and Jehee (2011) we showed that the assumption of multiplexing could reconcile timing and rate coding models. Here the focus is on the impact of bipolar codes on subtractive feedback.

Translating the learning algorithm to the more realistic context of separate signed inputs and synapses places additional demands on the neural circuitry, but also allows simpler interpretations of experimental observations. Our principal results are twofold. First, the push-pull characterizations of the feedforward and feedback pathways (Murphy et al., 1999; Hirsch, 2003; Martinez et al., 2005) are both direct consequences of emergent connectivity driven by a single algorithm for receptive field formation. This characterization is much simpler than other experimental descriptions in that a single cause explains both feedforward and feedback observations. The second result is that, in the feedback circuit learning model, the synaptic “weights” regularly change sign. The consequences for neurobiology are that synaptic contacts must be made or retracted. While the fact of synaptic growth and retraction is well established from experiments (Smythies, 2002; Trachtenberg et al., 2002; Stettler et al., 2006; Bourne and Harris, 2007, 2008; Yamahachi et al., 2009), we demonstrate how often it happens in the context of an algorithm for receptive field formation that monitors the synapse changes quantitatively throughout the receptive field formation process. The result is a prediction that the rate of change of new connections should be inversely correlated with the progress of receptive field formation.

## Materials and Methods

The overall methodology has been developed in earlier papers (Jehee et al., 2006; Jehee and Ballard, 2009). The novel contributions of this paper are to (1) develop an explicit formalism for characterizing the consequences of sign changes and (2) use this formalism in the simulations to track synapse changes in the circuits.

**Figure 1:**
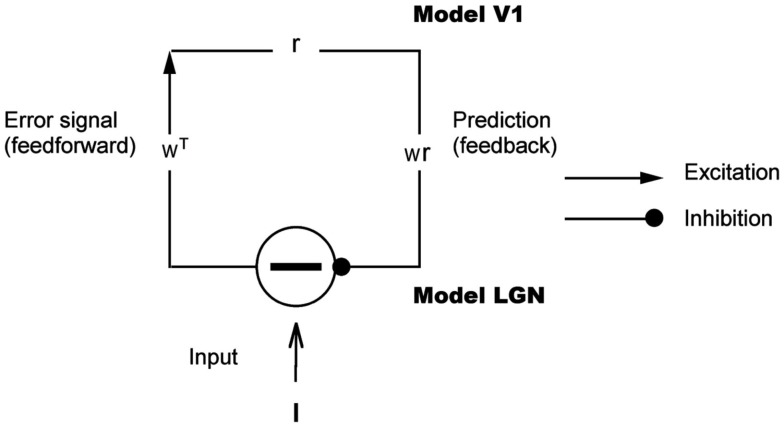
**Hierarchical model for predictive coding**. Higher-level units attempt to predict the responses of units in the next lower level via feedback connections, while lower-levels signal the difference between the prediction and the actual input. Feedforward connections encode the synaptic weights represented by a matrix *W ^T^*. Higher-level units maintain the current estimate of the input signal **r** and convey the top-down prediction *W***r** to the lower level via feedback connections. Difference detectors compute the difference **I** − *W***r** between current activity **I** and the top-down prediction *W***r**.

The LGN-V1 circuit model consists of two layers shown by Figure 1. The first layer, which models the lateral geniculate nucleus, consists of ON-center type and OFF-center type units. Similar to geniculate cells, ON-center type units code for brighter stimulus regions and OFF-center type units code for darker regions. We assume that either the ON-center unit or its OFF-center counterpart coding for the same spatial location is active at any given time step in the model. The model’s next higher level, which corresponds to an orientation column in primary visual cortex, receives input from the model LGN through feedforward connections.

In each feedforward-feedback cycle of the model, the feedforward receptive field that best matches the input, or equivalently the most likely prediction, is selected with high probability. The selection is made on the basis of the projections of the image, seen as a vector onto the synaptic weights, also see as vectors. The inputs to the model are small image patches of size 8 × 8 pixels selected at random form a database of 16 images of natural scenes.

A specific projection between a specific input **I** = (*x*_1_,…, *x_n_*) and a neuron with synapses **w** = (*w*_1_,…, *w_n_*) can be expressed as a scalar β, i.e.

$$\beta =\overset{N}{\underset{i=1}{\sum }}\;x_{i}w_{i}$$

or equivalently as the dot product β = **I**·**w**. This expression has some recent experimental evidence (Araya et al., 2006a,b). Variations impose some non-linearity on the result, e.g. (Pillow and Simoncelli, 2006; Sharpee et al., 2006).

Once a neuron with weights **w** is chosen on the basis of its projection, the learning rule moves it a little closer to the input vector, i.e.

$$\Delta \text{w}=\alpha \left(\text{I}-\beta \text{w}\right)$$

where β is the cosine of the angle between **I** and **w**. Note that by grouping the responses of all the neurons into a vector **r** (of which most of the components will be zero), one can summarize all the individual values β**w** as *W***r**, as is done in Figure 1. The scalar α is a learning parameter that is set to $\frac{.05}{1+\frac{p}{1000}}$ where *p* is the index of the *p*-th input patch seen by the circuit. The feedback connections are initially set to random values but are learned during the course of being exposed to 10,000–20,000 image patches.

For each patch, after a particular coding neuron is selected, the process repeats for 12 times, each time using the residual

$$\text{I}\leftarrow \left(\text{I}-\beta \text{w}\right).$$

**Figure 2:**
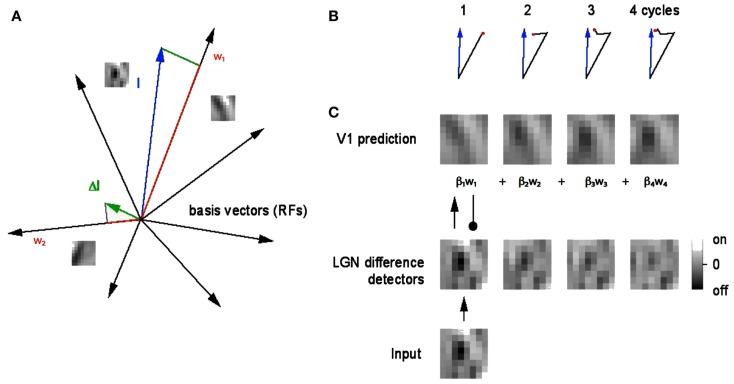
**A geometrical explanation of the matching pursuit learning model**. **(A)** An 8 × 8 filtered image patch can be represented as a blue vector with 128 coordinate values (in our notation). The neuron whose receptive field is most like the patch, in this case **w**_1_, is chosen to represent the patch. Since there are also 128 synapse strengths, or weights, these can be represented as a vector also. The difference between them is termed the residual (green) and is sent back to the LGN as feedback and the process repeats. A very small number of repetitions produces an accurate representation. **(B)** Four steps in the vector approximation. **(C)** The evolution of the approximation in pictorial terms. The green vector is also the basis for the learning algorithm. After each vector is chosen, it is moved closer to the input by adding the residual into its synaptic weight vector. The weight vectors are normalized to unity, reflecting a constraint that limits the total strength of the synapses.

A graphical interpretation of the algorithm is shown in Figure 2. The depicted geometry readily translates into two kinds of synapses. Feed forward synapses represent the input to V1 coding cells (or the residual of the input) and the feedback synapses represent the inputs (in the form of residuals) to LGN cells.

Both of these synapses can use a Hebb rule implemented as spike timing dependent plasticity (STDP) to make their synapses more like their input. In the feed forward pathway, the input on the first cycle will be the image, and the corresponding synapses are to be more image-like. Adding the image values to the synapses and normalizing accomplishes this. For the feedback pathway, considering the straightforward case of inhibition, if the cell fires, the feedback synapse needs to be increased proportional to the difference between the cell’s input and the feedback signal. Here again the synapses are made more like the input.

In our model we assume that every spike represents a scalar value. There is emergent evidence that this can be achieved by a latency code, where the time from spike arrival to a reference signal is the information carrier (VanRullen and Thorpe, 2002; Womelsdorf et al., 2007; Gollisch and Meister, 2008). Spikes can use a gamma band frequency and communicate values by using small (<5 ms) delays with respect to the reference. In a recent paper we simulated a spike latency code in some detail (Ballard and Jehee, 2011) to test its statistical properties, but here we assume that the gamma reference code is implicit and just use positive numbers and a clocked update process.

It has been established for some time that in a system like ours, the convergence of the feed forward and feedback pathways, where both use Hebb rules, is guaranteed. Furthermore the two sets of synapses, visualized together as a matrices, are such that the feed forward and feedback matrix values in the converged state are transposes of each other (Williams, 1985). Exactly how a neuron would implement a Hebb rule is not completely settled at this point, but our algorithm assumes that a STDP form of the rule, implemented at every spike cycle, is appropriate. Because of Williams’s result, in our simulation we take the shortcut of focusing on the feedback synapses and clamp the feed forward synapses to be the appropriate transpose of these.

The main contribution of the paper is to show that, using bipolar representations, the feedback signal requires six separate feedback path ways. In the algorithm, for any particular learning update, we test for which of these cases causes a spike and update only the synapses for that case accordingly. Since the other synapses do not produce spikes, and we assume an STDP form of Hebb rule, there is no need to update them.

**Figure 3:**
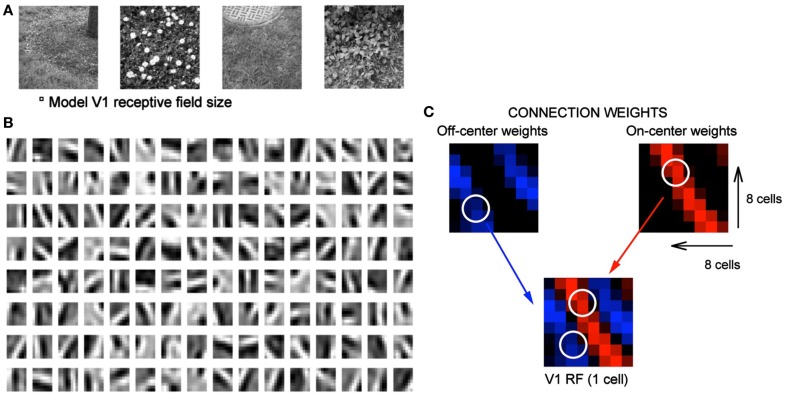
**Learning receptive fields with signed labeled lines**. **(A)** Subset of natural images used for training. The small square immediate below denotes model V1 receptive field size. **(B)** V1 receptive fields after training where ON and OFF responses are combined to produce a gray scale image. Black depicts off-regions in the model V1 receptive field, white depicts on-regions. **(C)** A detail from the feed forward connections in the model making the connections of different sign explicit. Blue denotes OFF-center connections and red denotes ON-center connections.

Our previous work (Jehee and Ballard, 2009), that learned the feed forward connections from cells in the LGN to a single cell in V1, is depicted in Figure 3. The learning algorithm connects a complete set of 128 synapses to the V1 cell initially, half from the ON calls and half from the OFF cells. However after learning only the appropriate set of LGN cells have large weights as shown in the figure. This replicates the experimental finding of Alonso and Reid (Reid and Alonso, 1995). They used antidromic simulation in paired recordings to confirm this connection arrangement. The experimental finding is very significant since it confirms the original suggestion that the connections could be formed in this manner by Hubel and Wiesel. What our simulation shows is that a Hebbian learning rule based on sparse coding principles is able to produce this arrangement.

To characterize these receptive fields, feedforward connection weights from ON-center type and OFF-center type LGN cells coding for the same spatial location are summed for each of the model’s 128 V1 cells. These summed weights are shown in Figure 3B. After training, the receptive fields show orientation tuning as found for simple cells in V1.

The model does retain the all-important feature of separate ON and OFF cells and, as a consequence, important structure emerges. The feedforward connections to simple cells respect the simple cells’ receptive field (Alonso et al., 2001) and the feedback connections from a simple cell target the appropriate LGN cells (Murphy et al., 1999). Both of these properties are observed as a result of the learning process in our model. The lower right portion of Figure 3 shows the detailed connectivity between 16 LGN cells and one simple cell after training. Here the color blue codes the synaptic strengths between OFF cells and red is used to code for synaptic strengths between the ON cells and the simple cell. What the figure shows is that for a representative learned receptive field, all the LGN cells that connect to it from an 8 × 8 array of OFF cells and an 8 × 8 array of ON cells connect to the appropriate part of the V1 cell’s receptive field with the appropriate synaptic strength.

One important difference between the model and the cortical circuitry is that the model agglomerates what are known to be many intermediate connections. Thus the LGN input to V1 terminates in layer IV and the feedback connections to the LGN originate in layer V and VI. However this important distinction is glossed over in the model which just has its LGN cells reciprocally connected to V1 cells. Furthermore our model uses cells that can have both excitatory and inhibitory synapses, even though this is not possible biologically. The understanding is that to produce inhibition, there must be an intermediate stage where the excitatory connection excites an inhibitory cell and vice versa. Rather than complicate the circuit diagrams, we allow model cells to have both kinds of connections.

## Results

The main result is that when using feedback that subtracts quantities, in specific synapses that are modified depend on the relative values of the minuend and subtrahend and that there are six distinct cases to consider. Furthermore, we show that, depending on the individual cases that arise, the modified synapses may be on different neurons. Subsequently we show in computer simulations that all six cases are need to handle natural image data and in the process of modifying synapses, the polarity of the needed connection may change, thus requiring a new connection. Tracking the rate of change of polarities shows that it is correlated with the progress of receptive field formation.

### Signed labeled line notation

We start by developing a notation for specifically denoting the relevant synapses. The response of a V1 cell can be characterized mathematically in terms of a function of the inputs multiplied by synaptic “weights,” that are numbers representing the strength of a synapse. Thus if the input to such a cell is represented by a vector *hbfx* and the synapses as a vector **w**, the response β can be given by

(1) $$\beta =f\left(\text{x}\cdot \text{w}\right)$$

where *f* is a function that captures any non-linearities in the response and **w**·**x** is the projection of **x** onto **w** or equivalently, the dot product between **x** and **w** given by

$$\text{w}\cdot \text{x}=\overset{N}{\underset{i=1}{\sum }}\;x_{i}w_{i}$$

**Figure 4:**
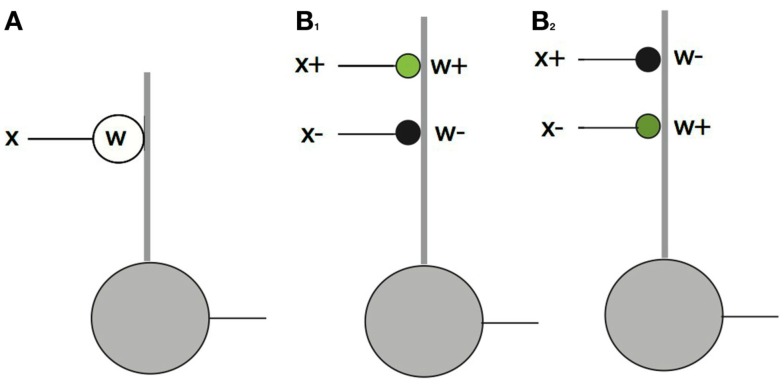
**The feed forward pathway connections**. The dot product computation illustrates the difference between conventional neural models and signed labeled lines. In the diagram thick lines denote dendrites and thin lines denote axons. *Green* circles = excitatory synapses. *Black* circles = inhibitory synapses. **(A)** If synapses and inputs could change sign then they could be handled simply with a single contact. **(B)** In the actual case there are four possibilities, each of which needs a separate synapse. Only one of *x*^+^*w*^+^ and *x*^+^*w*^−^ can be non-zero at any one time and the same holds for *x*^−^*w*^+^ and *x*^−^*w*^−^. Complementary pairs are required be non-zero to faithfully represent a dot product as shown for the cases of **B**_1_: *x*^+^*w*^+^ > 0 and **B**_2_: *x*^−^*w*^+^ > 0.

While the above expression models neuronal responses, and has experimental support for at least excitatory synapses (Araya et al., 2006a,b), it is cast at a level of abstraction that avoids the crucial issue associated with labeled lines and that is the representation of positive and negative coefficients. Let us illustrate these issues with the example of a single term in the expression in equation (1) above. Suppose a neuron is receiving input at a single synapse that can be expressed mathematically as the scalar product *wx*. Figure 4A shows how this multiplication would be implemented with signed quantities. Both the axonal input *x* and the synaptic strength *w* can be signed quantities, so a single “synapse” suffices to represent the calculation. However in the more detailed model that respects the representation of positive and negative quantities by separate cells the calculation cannot be done so easily. Besides the separate inputs, a further complication (from the standpoint of mathematical operations) is that biological synapses cannot change sign. An inhibitory synapse cannot become excitatory and vice versa.

Let us explore this complication in detail. The input *x* can be either positive or negative, denoted with {*x*^+^, *x*^−^} as can *w*, denoted with {*w*^+^, *w*^−^}. Thus to compute the product, four connections are required, representing all the combinations of positive and negative signs. Figure 4B shows these possibilities. Note that the figure is still a level of abstraction above the biological implementation of this relationship since a given set of synapses from any one neuron can only be excitatory or inhibitory. Thus at least one additional cell is required to change the inhibition to an excitation of an inhibitory cell. Note also that the ± notation is an *algebraic* device for keeping track of opponent quantities. For example *w*^+^ denotes the strength of a synapse. Whether or not it turns out to be excitatory or inhibitory depends on circuit and algorithm details. In the feedforward pathway *w*^+^ is positive and *w^−^* is negative, but in the feedback pathway they have to vary in sign.

When using signed labeled lines, the realization of elementary operations is not so straightforward and requires some care. To see this it helps to develop a notation for signed labeled line vectors. In standard vector notation, an example of a vector with two components is: $\text{x}=\left(\begin{array}{c} x_{1}\\ x_{2} \end{array}\right).$ A simple example showing the subtraction of two vectors is shown as follows

$$\left(\;\begin{array}{c} 1\\ -2 \end{array}\right)-\left(\;\begin{array}{c} 3\\ 1 \end{array}\right)=\left(\;\begin{array}{c} -2\\ -3 \end{array}\right)$$

This is standard vector mathematics, but now let’s introduce a convention that allows us to keep track of the fact that positive and negative components are represented by different cells. To express the subtraction in terms of labeled line notation, let’s use separate components for each of the positive and negative sides, as illustrated in the dot product example. Thus

$$\text{x}=\left(\;\begin{array}{c} x_{1}^{+}\\ x_{1}^{-}\\ x_{2}^{+}\\ x_{2}^{-} \end{array}\right)$$

and the above example becomes

$$\left(\;\begin{array}{c} 1\\ 0\\ 0\\ 2 \end{array}\right)-\left(\;\begin{array}{c} 3\\ 0\\ 1\\ 0 \end{array}\right)=\left(\;\begin{array}{c} 0\\ 2\\ 0\\ 3 \end{array}\right)$$

Note that each vector component must be either positive or negative so that in the labeled line notation, one of the two corresponding pairs is always zero. Furthermore note that in subtracting two vectors the result can be arbitrary in the sense that the resultant component that is non-zero depends on the signs and magnitudes of the vectors.

### Feedforward projections

In Figure 3 all the feed forward connections from the LGN to the model V1 cell are trying to make that cell produce a spike, that is they are all excitatory connections. What about inhibitory connections? Reid and Alonso (1995) showed that ON and OFF cells that did not connect to the appropriate parts of the V1 receptive field did not make excitatory connections, but there remains the possibility that they may make, by some route, inhibitory connections. Our model suggests that indeed this should be the case, and why by using our notation for signed labeled lines.

A basic step in the model is to compute the projection

$$\overset{N}{\underset{i=1}{\sum }}\;x_{i}w_{i}.$$

In terms of our new notation this can be rewritten as

$$\overset{N}{\underset{i=1}{\sum }}\;\left(x_{i}^{+}w_{i}^{+}+x_{i}^{+}w_{i}^{-}+x_{i}^{-}w_{i}^{+}+x_{i}^{-}w_{i}^{-}\right)$$

but since all the inputs are treated identically, let’s just concentrate on one such input and drop its subscript, so that the focus is on

$$x^{+}w^{+}+x^{+}w^{-}+x^{-}w^{+}+x^{-}w^{-}.$$

where in this case *w*^+^ is an excitatory synapse and *w^−^* is an inhibitory synapse. Taken at face value, this implies that there are four possible synapses that could be constructed to represent all the different possibilities for a term in the original dot product as shown in Figure 4. However when the receptive field is formed, on any cell, ideally only one of (*x*^+^*w^+^,x*^+^*w*^−^) should be non-zero. Furthermore the desired term in the dot product is positive and that can result from either *x*^+^*w*^+^ or *x*^−^*w*^+^ but not both. This results because, given the learning algorithm, in the dot product the positive synapse must track the time average <> of either <*x*^+^> or <*x*^−^>.

Let’s assume that the positive term that maximizes the projection is *x*^+^*w*^+^. Then for the projections to be calculated correctly, there needs to be a subtraction for the incorrect input *x*^−^. Thus the synaptic connection *x*^−^*w*^−^needs to be included where |*w*^−^| = |*w*^+^|. If it is not included, then inputs that should be discounted will not be, and as a consequence, those inputs will be recorded as better matches to a neuron’s receptive field than is in fact the case. This connection implies that feed forward inhibition should be anti-correlated as first argued by Miller (2003). As a side note, this inhibition can be handled in at least two ways. Either (1) the four connections can be present at a single cell, as depicted in Figure 4, or (2) two cells can be used one collecting *x*^+^*w*^+^ and the other collecting *x*^−^*w*^−^ followed by each cell laterally inhibiting the other. Lateral connections between simple cells are known to exist but the specificity implied by the need to represent the dot product correctly has not been established. Nonetheless a prediction of the signed labeled line model is that this specificity has to appear in some form of which the two possibilities just discussed are the prime candidates and there is evidence for both (Hirsch, 2003; Martinez et al., 2005; Wang et al., 2007).

This notation allows the tracking of sign changes but has one drawback. In the feedback connections, it turns out that when using feedback, a *value* such as *w*^+^ sometimes can be the weight of an *inhibitory* connection, and vice versa. The diagrams make clear whether inhibition or excitation is intended.

### Feedback projections

The algorithm elaborated upon in the Materials and Methods Section represents input by rapidly and sequentially selecting a handful of neurons to represent it. The algorithm is conceptually simple: one of the neurons that best matches the input is selected first, then that neuron’s contribution is subtracted from the input via a feedback signal with the result that the remainder is in the form of new input and the process is repeated. However handling negative feedback in the signed labeled line system is far from straightforward and must be handled on a case by case basis. As will be demonstrated, the net result is that the different cases need to be realized in separate circuitry. To illustrate the signed labeled line solution, consider the central calculation of the matching pursuit circuit described graphically in Figure 9. In the feed forward pathway the projection of the input onto the largest vector must be calculated. The result is given by **x**·**w**_1_ in standard notation and we have termed this quantity β. The feedback is given by the difference between the input vector **x** and its projection β into the closest vector described by its synapses. Where **w**_1_ is the closest such vector, this difference is given by:

$$\text{x}-\beta {\text{w}}_{1}$$

Note that the need to deal with subtraction is a central requisite of this algorithm but of course not specialized to it. Any algorithm that required subtraction will have this issue.

**Figure 5:**
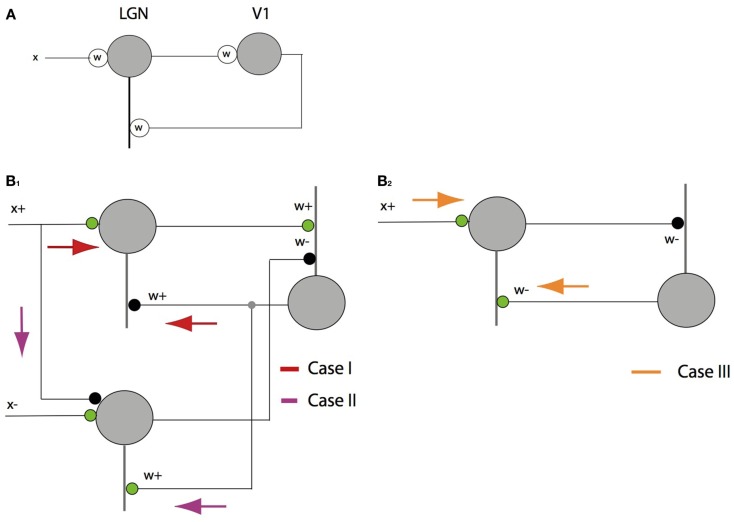
**The feedback pathway connections**. **(A)** An inhibitory feedback circuit is simple to describe with signed synapses and signals. **(B)** Special care must be taken when using signed labeled lines. Analyzing the feedback to a single cell requires treating *x*^+^ separately from *x*^−^, but here only the three cases for *x*^+^ are analyzed as the cases for *x*^−^ are symmetric. **(B_1_)** Case I: in the simplest to understand case *x*^+^ > β*w*^+^ > 0, the feed forward circuit computes the projection β and the feedback component is inhibitory. Case II: when *x*^+^ < β*w*^+^, things are more complicated as the feedback must *excite* the complementary LGN cell. **(B_2_)** Case III: when *w*^−^ > 0 the feedback is excitatory also but to the *x*^+^ cell.

Since all the components of the vector are treated identically, for simplicity of both notation and exposition, again we will focus on just one vector component. Thus in the subsequent calculations all the variables are scalars. The difference between the input and vector projection for a single component can be indicated by *x* − β*w* in standard notation. In signed labeled line notation this becomes

$$\left(\;\begin{array}{c} x^{+}\\ x^{-} \end{array}\right)-\beta \left(\;\begin{array}{c} w^{+}\\ w^{-} \end{array}\right)$$

where of course only one of *x*^+^ and *x*^−^ can be non-zero at any one time. Similarly only one of *w*^+^ and *w*^−^ can be simultaneously non-zero. We illustrate the circuitry for *x*^+^ non-zero. The circuitry for *x*^−^ non-zero is handled symmetrically. When *x*^+^ is non-zero, there are a number of different cases, each of which require different circuitry. For each such case, we indicate the resultant circuit pathway with colored arrows, as shown in Figure 5.

$$\begin{array}{rcll} & \text{Case I: }{\text{x}}^{+}\;>\;\beta {\text{w}}^{+} & & \\ & \left(\;\begin{array}{c} x^{+}\\ 0 \end{array}\right)-\beta \left(\;\begin{array}{c} w^{+}\\ 0 \end{array}\right)=\left(\;\begin{array}{c} x^{+}-\beta w^{+}\\ 0 \end{array}\right) & & \end{array}$$

This is a simple case. The feedback pathway is inhibitory and has value *w*^+^.

$$\begin{array}{rcll} & \text{Case II: }{\text{x}}^{+}\;<\;\beta {\text{w}}^{+} & & \\ & \left(\;\begin{array}{c} x^{+}\\ 0 \end{array}\right)-\beta \left(\;\begin{array}{c} w^{+}\\ 0 \end{array}\right)=\left(\;\begin{array}{c} 0\\ \beta w^{+}-x^{+} \end{array}\right) & & \end{array}$$

This case is a little tricky but important. The result uses −*x*^+^. To realize this, *x*^+^ has to be fed into the negative side, i.e., the opponent neuron, with an inhibitory connection, and the feedback to that neuron has to be positive or excitatory. As shown by Jehee and Ballard (2009), this component of the circuit can be a form of rebound that introduces a temporal transient when the inputs are suddenly disturbed.

$$\begin{array}{rcll} & \text{Case III: }{\text{w}}^{-}\;>\;0 & & \\ & \left(\;\begin{array}{c} x^{+}\\ 0 \end{array}\right)-\beta \left(\;\begin{array}{c} 0\\ w^{-} \end{array}\right)=\left(\;\begin{array}{c} x^{+}+\beta w^{-}\\ 0 \end{array}\right) & & \end{array}$$

This is another simple case. The feedback pathway is excitatory and has value *w*^+^. By considering *x^−^*, the need for three more pathways can be demonstrated for a total of six overall.

With six parallel feedback pathways, a concern is whether they would interfere. A case by case analysis conforms that the circuit will function as desired. Let’s examine the *x*^+^ three cases. Case I does not interfere with Case II because when the values are appropriate for Case I, the circuitry on the complementary side is held off. Similarly when Case II is appropriate, the circuitry for Case I is held off by virtue of the relative values. As for Case III, when the synapse *w*^−^ > 0, its complement *w*^+^ is 0 so none of the circuitry is needed. Thus the *x*^+^ feedback pathway is different depending on whether <*x*^+^> or <*x*^−^> dominates. If the former, then the feedback circuitry should look like Figure 5B_1_, if the latter then the feedback circuitry should look like Figure 5B_2_. With regard to this analysis, there are a very nice synergy between the latency coding and STDP. In both STDP and our model, if the net input to a cell is not sufficient to make it spike then the learning does not occur. Thus the signal can be sent down three pathways at a time, but only the correct pathway will produce a spike.

These relationships might be more complicated if the circuitry had to operate in parallel with multiple, simultaneous feedback pathways. However a fundamental property of the algorithm is that only one coding (V1) neuron is analyzed per iteration. Owing to this property, the cases hold for each of the model LGN neurons.

**Figure 6:**
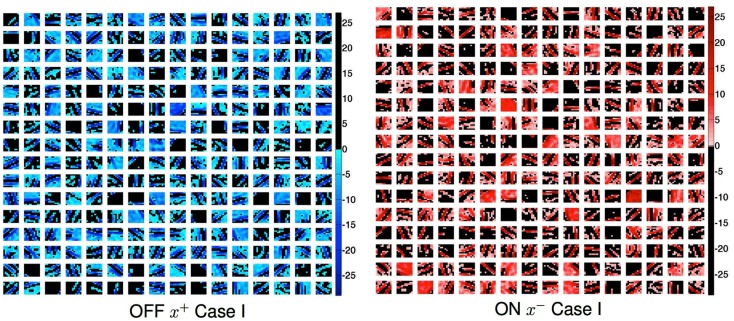
**Case I feedback connections from cortical cells to LGN OFF and ON cells obtained in a simulation of a larger system with 10 × 10 patches and 256 cortical cells**. The Each LGN cell has a coordinate in all the sub-matrices. If that coordinate is colored then it is receiving inhibitory input from the cortical cell represented by the sub-matrix. OFF: if the input connection to the LGN cell had an *x*^+^ input, i.e., was positive, then the primary feedback to the ON cell and will be negative. ON: if the feedforward connection came from an OFF cell (*x***^−^** input and there fore positive), then the primary feedback to that OFF cell and will be negative. Similar connection patterns can be generated for the other four cases, but with appropriately different connections and polarities as dictated by Figure 5. The color coding convention follows Figure 3.

Figure 6 shows the result of the learned synapses of 512 V1 cells connecting to 100 LGN cells. Experiments have shown that the feedback connections of cortical simple cells are inhibitory when the “ON” field of a simple cell feedback connects to its corresponding input ON LGN cell, and excitatory when it connects to the corresponding OFF LGN cell (Wang et al., 2006) and the Case I connections in our simulation replicate the experimental result.

**Figure 7:**
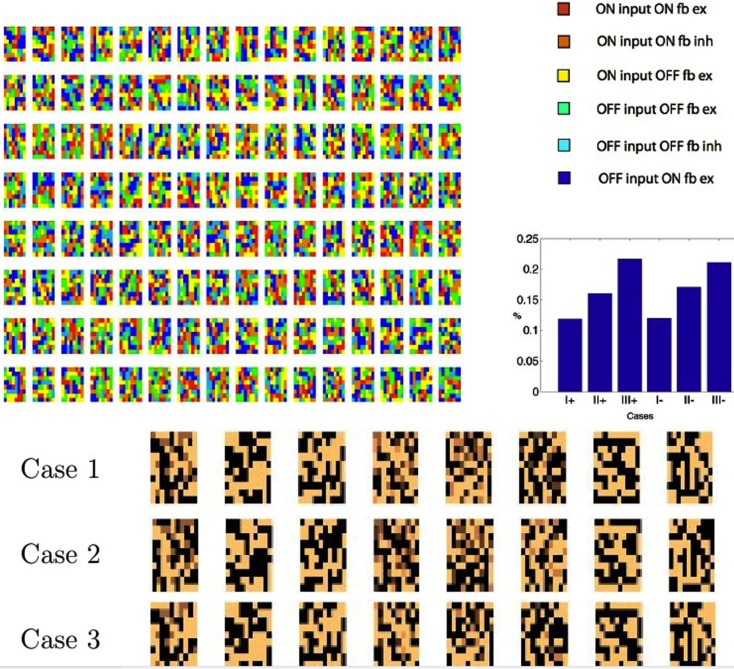
**Tracking instantaneous feedback routes**. Colors denote the six different routes that feedback can travel. Top left: for each model neuron, the last time it was chosen its feedback pathway for each of its synapses is labeled with a color denoting the route to each of the LGN neurons selected. The colors show that all six cases are realized. Top right: a histogram of the frequency of usage of the different cases. Bottom: a small segment of 32 applications of learning shows the beginnings of synapse learning. 10 **×** 10 maps of the combined connection strengths to the LGN cells are shown.

The analysis has revealed six separate cases but one can wonder whether they are all used by the algorithm. In other words, is image data such that some of the cases do not occur? The simulation conforms that all six cases are used. Figure 7 shows this result. Each time a V1 cell is selected, it must send feedback to each of the 8 × 8 × 2 LGN cells that it is connected to. For each of those cells, only one of the six cases will come up. For this reason we can create a color coded image with the rule that, for each V1 coding neuron, the last time it was selected, for each of its feedback targeted LGN neurons, we can color code the route that the feedback took. To unpack this explanation a bit more, realize that each of the positions in Figure 7 can represent any of the six possible pathways to the LGN at that location. The specific color displayed denotes, for a particular feedback moment in time, which of the six pathways was actually used. The colors in the top left of Figure 7 reveal that typically all six cases are present. Furthermore they are used extensively. Figure 7 (top right) shows a histogram of the routes over a large sample of cells. Figure 3 tracked the Case I feedback synapses, but all six cases learn. The bottom traces in Figure 7 shows the beginning of learning in all three cases for eight sets of LGN synapses from the three *x*^+^ cases. Synapses in all three cases have been updated. The Case II synapses are inhibitory.

### Tracking dynamic synapses

Research on the formation of synapses is showing that their formation and maintenance is very dynamic (Trachtenberg et al., 2002; Bourne and Harris, 2007, 2008; Yamahachi et al., 2009). Given that we can explicitly represent signs of synapses, one very important consideration is; Do the connections in the model circuitry need to change from one polarity to another? The consequences are significant because for example, if the dominant input to a coding cell changes from <*x*^+^> to <*x*^−^>, then the feedback pathway that was in the form of Figure 5B_1_ has to change somehow to that of Figure 5B_2_. The simulations show that such polarity changes are indeed the case; connections can be required to change from inhibitory to excitatory and vice versa throughout the learning process. In the model, all possible connections are present initially and just their strength is modulated by the algorithm. Perhaps quite naturally, in the course of learning their final values, the synaptic weights change sign fairly often. They start out by making many changes and then gravitate to making fewer changes in the final stages of synaptic convergence.

**Figure 8:**
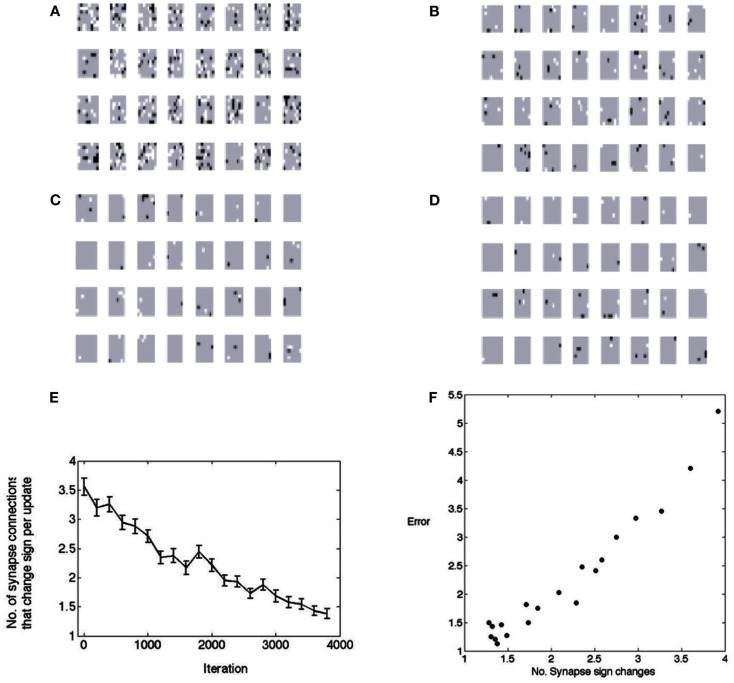
**The changes in polarity of receptive fields during learning**. A change from positive to negative is denoted by black and a change from negative to positive is denoted by white. **(A)** After the first 3,000 image patches. **(B)** After the second 3,000 image patches. **(C)** After the third 3,000 image patches. **(D)** After the forth 3,000 image patches. **(E)** The points plotted show the average number of synapses that had to change from a base of 256. Fifty samples are used in computing the standard error bars. Thus initially, on every learning update, about 5% of the synapses need to change signs. At the end of learning this number is down to less than 0.5%. **(F)** The change in synapse polarity is tightly correlated with the residual error in fitting receptive fields (*r* = 0.97), suggesting that the changes in polarity can be used to track the progress of receptive field formation.

Figure 8 shows this by testing the polarity of the weights every 3,000 image samples. As is evident, a large fraction of the synapses change their values. During the first 3,000 iterations about 4,000 of the total of 8,192 feedforward synapses change their values. If they are not needed they drift toward zero, but if they are needed an excitatory contact may have to be replaced by an inhibitory contact or vice versa. The figure shows the change from excitatory to inhibitory as black and the opposite change as white. Most of the changes are in the early stages, but the synapses can change even near the end of the learning process. The model is non-committal as to how synapse changes are accomplished. The synapses need to change throughout the learning process, but the number decreases to less than 0.05% per learning rule update (an update refers to the selection of a neuron in the matching pursuit process – see Materials and Methods). However at the beginning the rate of sign changes may seem low at 5%, but remember that this is for each neuron that is selected, so in fact the cortical connection process needs to be very dynamic. What perhaps might have been expected, but nonetheless is very interesting to observe, is that the progress of receptive field formation is highly correlated (*r* = 0.97) with the number of polarity changes, as shown in Figure 8F. This hints that the rate of polarity change could be a highly informative developmental measure.

**Figure 9:**
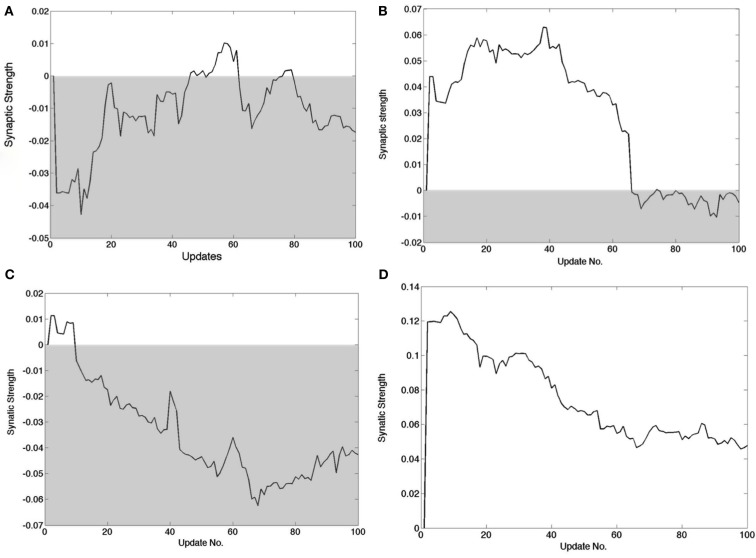
**Detailed tracks of the feed forward synapse to the cortical cell during the learning process**. The shaded area delimits the region wherein the connection is inhibitory. The synapse changing sign signifies that the *B*_1_ connections have to change to those in *B*_2_ in Figure 5. **(A)** A synapse that if briefly positive in two instances but ends up negative. **(B)** A synapse that is positive for most of the learning process but ends up slightly negative. **(C)** A synapse that starts out positive but very soon becomes negative and remains so. **(D)** A synapse that decreases in strength but is always positive.

Figure 8 summarizes synaptic changes but it is of interest to track the dynamics of synapses in detail. In the learning algorithm, every time a cortical neuron is chosen to represent a signal, a small adjustment is made in the synaptic strengths. Corresponding adjustments have to be made in the feedback pathway. Although the amount the synaptic strength changes in each update is a user-set parameter, it is still instructive to chart the progress of the synapses as a function of updates, as shown in Figure 9. Four feed forward synapses coding the *x*^+^ signal from a randomly selected cortical neuron reveal very different dynamic trajectories. However each time one of these synapses changes polarity from positive to negative, the circuitry representing *x*^+^ must switch from the Case I/II configuration to the Case III configuration and vice versa.

**Figure 10:**
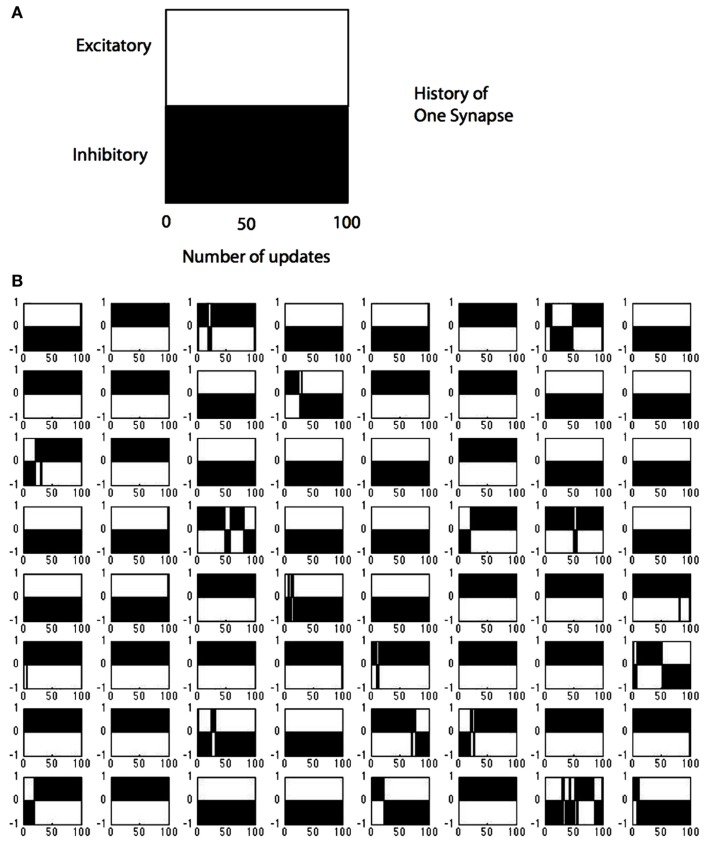
**Tracking the polarity of connections to a single cell**. **(A)** The black portion of the legend denotes the polarity of a synapse (in this case inhibitory) as a function of the number of updates. **(B)** The state of all 128 possible synapses for a particular model simple cell as a function of the times that it was chosen to represent an input image stimulus. The figure represents two possible synapses at each location. Most locations can be represented by a single synapse that does not change sign, but at 19 positions the synapses need to change sign during the computation, some several times such as synapse position (8,7).

The sign changes for all the synapses in a single V1 cell are summarized in Figure 10. Each time any neuron is updated the number of sign changes in its synapses are recorded and the model allows us to inspect these changes. To demonstrate this capability, we track the behavior of model neuron #54 (out of 128) in Figure 10 which shows the course of each of its 64 synapses. The *x*-axis records the updates, that is each time that particular neuron was selected for modification (in the course of the learning algorithm there were intervening periods where other neurons were chosen). The simulation data for model neuron #54 shows that for the first 100 updates, 19 of 64 synapses changed from one polarity to another. For example synapse location (4,3) started out as excitatory (+1), switched to inhibitory (−1) around update 50 and then switched back to excitatory and finished as an inhibitory connection. By comparison, synapse (4,4) was always inhibitory and synapse (1,6) was always excitatory.

## Discussion

Most neural network simulations ignore the detailed constraints of real neurons, blithely assuming that synapses can change signs and that huge precision is available in the intracellular signaling. The assumption is that the mathematics is important and the implementation issues are just unimportant details. Our simulations support this by showing that when these details are taken into account, the results that have been obtained with the more abstract models do indeed extend to the more detailed setting. However the labeled line model provides an important new vista into neural coding of dynamical circuits in several aspects.

### Multiple, separate feedback pathways are required

Surprisingly, from the standpoint of the model its feedback travels along different pathways depending on whether it is negative or positive. This is at least testable and may have already been tested. Sillito et al. (Murphy et al., 1999) has observed that cortical feedback to the LGN is phase reversed, meaning that if the cortical simple cell connects back to an LGN cell of the opposite polarity as measured with respect to the cortical cell’s receptive field, then that connection is excitatory. They recognize that this is a push-pull circuit, and speculate on its function as “gain control and linearity in the transfer of input to the cortex,” but from our perspective a potential function is much simpler. The phase-reversed connections occur as Case II of our signed labeled lines feedback, and thus are a direct consequence of an algorithm, which is trying to represent stimuli in an economical way and compute synapse strengths via negative feedback.

### Excitatory and inhibitory synapses are exchanged in RF formation

Learning in the labeled line model can require that an excitatory synapse be replaced by an inhibitory one and vice versa. This means that these synapses must be coupled somehow, so that the state of one can be available in some form to its complement. The simulations herein do not address the mechanism for accomplishing this but it needs to be done. This observation is not as evident from signed representations. Furthermore the number of synapses that have to change sign from iteration to iteration is substantial, being about 3%, and, if there were a way of measuring synaptic dynamics *en mass*, this could be tested.

One issue that is not simple to explain is that the synapses can be set with so few updates. After about 400 updates per model neuron the synapses have converged to their final values. Given that an update in our simulation might only take 20–100 ms, it is hard to explain why the biological process seems to take much longer. One way this could arise is if there were overhead in setting up the synapses in the first place; our model does not represent this difficulty. Another slowdown factor might be that the amount a synapse can change per update is much less than assumed by the model. In any case the model provides the beginning of a processes of simulating alternate hypotheses.

### Hebbian learning rules

Learning is also impacted by the signed labeled line representation. Although the rules are Hebbian in that they are local, their actual implementation must be different in the feed forward and feedback pathways. In the feed forward pathway the desired modification is carried by an error signal in the spike code itself whereas in the feedback pathway the modification is a result of the difference between the feedback and other inputs. An additional complication is that, owing to the fact that, for each model neuron, there is no signal for one or other of the polarities, only one side of the correction can be actively implemented. This makes the signed labeled line learning system compatible with spike timing dependent plasticity (STDP; Bi and Poo, 1998; Dan and Poo, 2004), but, in that scheme negative corrections to synaptic strength are signaled with spike timing advances, a feature that is not used by our model. One implication is that STDP plays some additional roles in the management of cortical circuits.

### Signed labeled lines make squaring simple

Some abstract models of motion detection require a squaring function to overcome the fact that while the signed signal may be uncorrelated, its absolute value is usefully correlated (Simoncelli and Heeger, 1996). However, just how does neurobiology come up with such a function? In the labeled line representation, this is much less of a problem than in signed representations as the signal is easily rectified by treating the “negative” part of the signal as positive.

### Fitting the time course of learning to data

The model makes the intriguing prediction that the number of changes in synaptic polarity over time tracks that progress of receptive field formation but does not ground this progress in terms of observed time courses in neural development. However it may be that the observed progress of visual perception in humans can serve to bracket a possible correspondence. The ability to perceive kinetic depth occurs in the first months of development and stereo depth perception occurs at about 4 months (Yonas and Aterberry, 1987). Thus in terms of early visual cortex the predominant changes should be observed with a time constant of a few months.

## Conclusion

New techniques that allow the elucidation of the details of cortical circuitry are showing that the cortical matrix of cells is very detailed (Yoshimura et al., 2005; Nassi et al., 2006; Luo et al., 2008) and under considerable genetic control (Cubelos et al., 2010), so to decipher it, it is likely that all useful constraints will need to be brought to bear. We show here that the interaction of a standard algorithm with the basic cortical coding of signed information can explain experimental observations of push-pull circuitry in both feed forward and feedback pathways.

An important point to note is that, even at its chosen level of abstraction, the feedback circuit is not the only way of modeling the formation of receptive fields by learning natural image statistics. Spratling (2008) has shown that feedback can equivalently modeled by lateral inhibition at the cortical level, and which of these two methods are used will have to be settled experimentally. However wherever subtraction is used in a circuit with signed labeled line encodings, the issues addressed in this paper will arise.

It is possible to design model circuits that use division (divisive normalization) instead of subtraction. The model by Ozeki et al. (2009) sheds light on the constraints governing the cortical implementation of inhibition, but at the same time sidesteps larger issues tackled herein such as learning receptive fields. The design by Spratling (2010) provides an excellent fit to a very large number of experimental observations, and has recently been extended with learning algorithms (Spratling, 2012) for its synapses. In comparison, the learning algorithm used here is a variant of that of Jehee et al. (2006) and has a formal Bayesian grounding. Furthermore Ballard and Jehee (2011) show that it has a potential explanation of the observed Poisson randomness observed in cortical spikes. The simulation of the learning algorithm here shows that it is still effective, despite the overhead produced by a bipolar coding strategy.

A final important thing to keep in mind is that although the model is much more detailed than the majority of neural models that used signed representations for synapse and neuronal outputs, it is still very abstract in that it ignores many of the still more detailed aspects of cortical architecture (Reichova and Sherman, 2004). This architecture is obviously used for many functions in the course of implementing complex behaviors and those functions must be represented in additional circuitry to that assumed by our model. Furthermore it is well known that the feedback loop from striate cortex to LGN is complicated by many intermediate connections. For example the input to striate cortex terminates in layer IV whereas the output to the LGN originates from layers V and VI. In our model this complexity is summarized in single model neurons that receive both input and provide output. Along these lines there is another area in the simulation would need to be refined, and that is the fact that in the cortex the number of excitatory synapses outnumbers the number of inhibitory synapses. One estimate (Sherman and Guillery, 2003) is that the ratio of excitatory synapses to inhibitory synapses is on the order of 84:16. Since the ratio in the model is very close to 1:1, this means that there must be a pooling of inhibition where by multiple network inhibitory connections are handled by registering them as excitatory on an intermediate cell that then has a single inhibitory connection of the net value on the original destination cell.

Cortical circuitry is extremely complex, and in order to completely understand and integrate its structure and function, a wide variety of data from different sources will have to be synthesized into a coherent picture. In that effort, the constraints in this paper, which relate the dynamics of synapse formation to both the basic cortical representation of signals and a promising algorithm for understanding the representation’s formation, may turn out to be a very important component.

## Conflict of Interest Statement

The authors declare that the research was conducted in the absence of any commercial or financial relationships that could be construed as a potential conflict of interest.
